# Deformation mechanism of innovative 3D chiral metamaterials

**DOI:** 10.1038/s41598-018-30737-7

**Published:** 2018-08-22

**Authors:** Wenwang Wu, Dexing Qi, Haitao Liao, Guian Qian, Luchao Geng, Yinghao Niu, Jun Liang

**Affiliations:** 1 Institute of Advanced Structure Technology, Beijing Institute of Technology, Beijing, 100081 China; 2 Beijing Key Laboratory of Lightweight Multi-functional Composite Materials and Structures, Beijing, 100081 China; 3 Laboratory for Nuclear Materials, Paul Scherrer Institute, Villigen PSI, 5232 Switzerland; 4 State Key Laboratory for Turbulence and Complex Systems, College of Engineering, Peking University, Beijing, 100871 China

## Abstract

Rational design of artificial microstructured metamaterials with advanced mechanical and physical properties that are not accessible in nature materials is very important. Making use of node rotation and ligament bending deformation features of chiral materials, two types of innovative 3D chiral metamaterials are proposed, namely chiral- chiral- antichiral and chiral- antichiral- antichiral metamaterials. *In-situ* compression and uniaxial tensile tests are performed for studying the mechanical properties and deformation mechanisms of these two types of 3D chiral metamaterials. Novel deformation mechanisms along different directions are explored and analyzed, such as: uniform spatial rotation deformation, tensile-shearing directed (compression-shearing directed), tensile-expansion directed (compression-shrinkage directed) deformation mechanisms of 3D chiral metamaterials, and competitions between different types of deformation mechanisms are discussed. The proposed 3D chiral metamaterials represents a series of metamaterials with robust microstructures design feasibilities.

## Introduction

Rational design of artificial micro architected metamaterials with advanced mechanical and physical properties that are not accessible in nature materials is challenging and important. Artificially designed metamaterials are usually arranged in repeating patterns, at scales that are smaller than the wavelengths of the phenomena they influence. The smart properties of metamaterials origin from artificially designed structures at sub-wavelength scales, and can be tailored and tuned precisely with their architected shape, geometry, size, orientation and arrangements. The interactions between architected microstructures of metamaterials and electromagnetic, sound and optical waves will result in blocking, absorbing, enhancing, or bending of waves. It has been demonstrated that rationally designed metamaterials have promising multifunctional applications, such as ultralow mass densities and ultrastrong metamaterials^1–5^, sound and vibration attenuation metamaterials^6,7^, electromagnetic cloaking metamaterials^8,9^, negative thermal expansion metamaterials^10,11^, subwave length optical metamaterials^12–15^, etc.

As a special type of mechanical metamaterials, auxetic metamaterials with negative Poisson ratio can expand its volume when stretched, and the concept of auxetic materials with negative Poisson ratio was firstly described by Love in 1944 for the first time^16^. Auxetic metamaterials exhibit enhanced mechanical properties over conventional materials, such as higher shearing modulus, increased indentation resistance, good absorption properties and higher fracture toughness. Auxetic materials can be applied for designing innovative multifunctional structures, such as: body armor, packing material, knee and elbow pads, robust shock absorbing material and sponge mops. According to the geometrical relations of auxetic unit cell, there are mainly three types of auxetic materials: reentrant materials, rigid square rotation materials and chiral structures^17^. Chiral structures stands for a series of structures which cannot be mapped onto its mirror image by rotations and translations alone^18^, and various types of chiral structures exist commonly in nature, such as: DNA, RNA, chiral carbon nanotube, twisting flower petals and stems, plant climbing tendrils and twisted leaves, chiral cellulose^19–21^. Besides these chiral materials in nature, various types of multifunctional artificial chiral metamaterials are designed and fabricated as well. Because of their lack of mirror symmetry, chiral metamaterials^22,23^ have recently enabled several remarkable phenomena, such as negative refractive index^24^, superchiral light^25^, and use as broadband circular polarizers^26,27^ or detectors^28^.

The mechanical properties of chiral structures can be investigated with different theoretical approaches, such as: strain energy homogenization method, internal external force equilibrium of unit cell, Cosserat (micro-polar) elasticity, etc^29–34^. In the classical theory of elasticity, the degrees of freedom are not included for describing the mechanical behaviors of microstructured solid where the microstructure characteristic length is comparable to the solid structure^35^. Due to the additional degrees of freedom allowed by internal microstructures, chiral Cosserat solids have different mechanical behaviors from solids with a center of symmetry^35–37^, The Cosserat (also called micropolar) elasticity theory of Eringen^37^ demonstrated robust efficiency and reliability in the modeling of materials with microstructures, such as: granular or fibrous materials, bone microstructure, or 3D lattice structures, etc. For example, based on the micropolar theory and tensor analysis, Liu *et al*.^38^ developed a continuum theory for describing the dilatation–rotation coupling and shear–rotation coupling deformation mechanism of 2D chiral lattice structures. Chen *et al*.^39^ proposed a micropolar continuum model for describing the constitutive relation for tetrachiral lattice structure, where 13 independent material constants are employed. Spadoni *et al*.^40^ proposed a micropolar continuum model for analyzing the in-plane properties of hexachiral structures, where deformable-ring node model are employed.

Recently, Kang *et al*.^41^ exploited the buckling introduced mechanical instabilities in surface-attached cellular structure, and effects of cellular unit cell geometrical parameters on the formed chiral pattern during the swelling and shrinkage cycle are studied systematically. Shan *et al*.^42^ proposed an elastomeric porous metamaterials, where multiple pattern transformations can be induced by buckling. The proposed periodic porous elastic structures can generate mechanical instabilities, and can be used to tune the propagation of elastic waves in phononic crystals, enhancing the tunability of the dynamic response of the system. Ha *et al*.^43,44^ proposed an innovative isotropic 3D tetrachiral metastructure, and studied its mechanical properties via finite element analysis. Fu *et al*.^45^ developed the equivalent modulus and Poisson ratio of a novel 3D chiral structures made up of orthogonal assembled 2D chiral honeycomb with four ligaments. Based on the pioneering work on “missing rib” type of chiral structures designed by Smith *et al*.^46^, new chiral cellular solids with center cores and softer hinges are designed and fabricated via multi-material 3D printing techniques, and amplified chirality-induced auxetic effect via elevating internal rotation efficiency can be realized^47^. Making use of the chiral rotation induced unique sequential cell-opening mechanisms, hybrid auxetic chiral mechanical metamaterial are designed, which can be employed for developing new multi-functional smart composites, sensors and/or actuators^48^. In order to overcome the twist deformation limits of linearly elastic bar, Frenzel *et al*.^49^ proposed a microstructured 3D elastic chiral mechanical metamaterials which can realize twist deformation upon compression, the proposed tension/compression induced twist deformation has potential applications in chiral optical metamaterials, such as: optical dynamic cloaking structures. Sha *et al*.^50^ proposed the design of large-scale chiral metallic glasses with extensive hardening and large ductility properties, the mechanical behaviors of the metallic glass chiral nanolattice (MGCN) can be significantly altered through changing the thickness and length of the ligaments in the nanolattices.

In this paper, depending on the geometrical relations between nodes and ligaments, two types of innovative 3D chiral metamaterials are proposed, namely chiral- chiral- antichiral, and chiral- antichiral- antichiral metamaterials. Firstly, two series of these two types of 3D chiral metamaterials with different geometrical parameters are designed, and *in-situ* compression tests are performed for studying the mechanical properties and deformation mechanisms of these two types of 3D chiral metamaterials. Novel deformation mechanisms such as: compression-shearing, compression-shrinking auxetic deformation of chiral unit cell, antichiral unit cell and chiral-antichiral hybrid unit cell along different directions are explored and analyzed. Secondly, *in-situ* uniaxial tensile tests are carried out, and competitions between different types of deformation mechanisms are discussed. With the progress of micro- and nano- manufacturing techniques, the proposed 3D chiral metamaterials show promising performances for future industrial applications, such as: sound absorption and vibration metamaterials, morphing structures, chiral optical metamaterials, shape memory actuators and biomechanical devices.

## Mechanical Properties of 3D chiral metamaterials

### Topological design of innovative 3D chiral metamaterials

**Figure 1:**
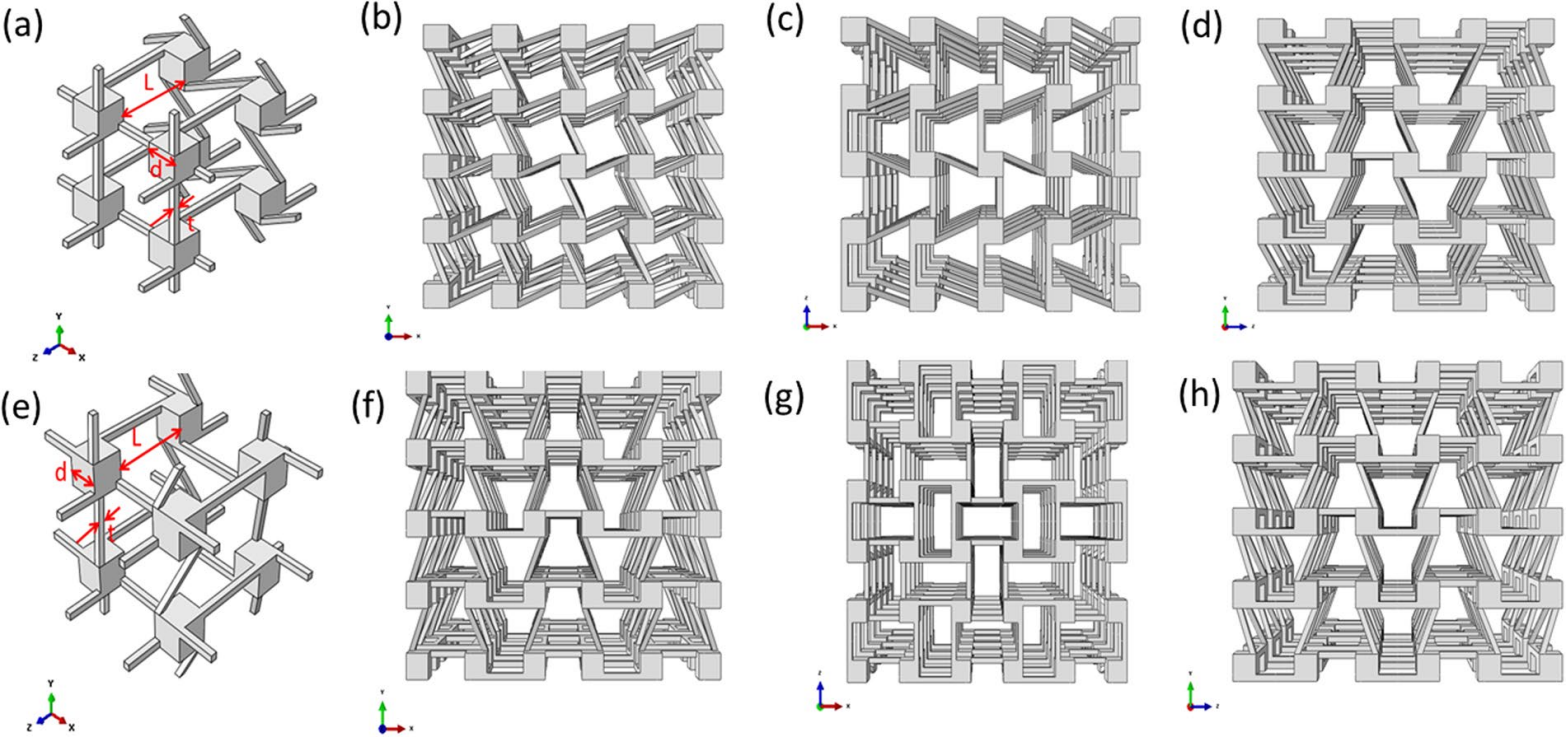
The x-y, y-z, z-x and stereo views of the architected 3D chiral matamaterials (**a**,**b**,**c**) and (**d**) chiral- chiral- antichiral metamaterials; (**e**,**f**,**g**) and (**h**) chiral- antichiral- antichiral metamaterials.

Chiral structures are architected with circular, polygonal, elliptical, sphere or cubic nodes and ligaments connecting neighboring nodes in 2D or 3D spaces, the deformation modes of chiral metamaterials are featured by node rotation and ligaments bending deformation under external loading conditions. Depending on the geometrical relation between ligaments and nodes of 3D chiral metastructures, there are chiral and antichiral topological configurations along x, y and z directions respectively. As shown in Fig. 1, two types of 3D chiral unit cells are proposed, namely chiral- chiral- antichiral and chiral- antichiral- antichiral architected metastructures, respectively. The x-y, y-z, z-x views and stereo views of these two types of metastructures are shown in Fig. 1(a–h), respectively. The topology layout of these two types of 3D chiral metamaterials can be realized in two steps: Firstly, constructing 3D chiral unit cell; Secondly, generating the global metamaterials through periodic distribution of 3D chiral unit cells along the x, y, z directions. As shown in Fig. 1(a,e), the geometrical parameters for describing the 3D chiral unit cells with cubic nodes are: ligament perpendicular length *L* along x, y and z directions, cubic node side length *d*, ligaments thickness *t*, the numbers of unit cells along x, y and z directions are *N*_*x*_, *N*_*y*_ and *N*_*z*_ respectively.

### *In-situ* compression tests of 3D chiral metamaterials

**Figure 2:**
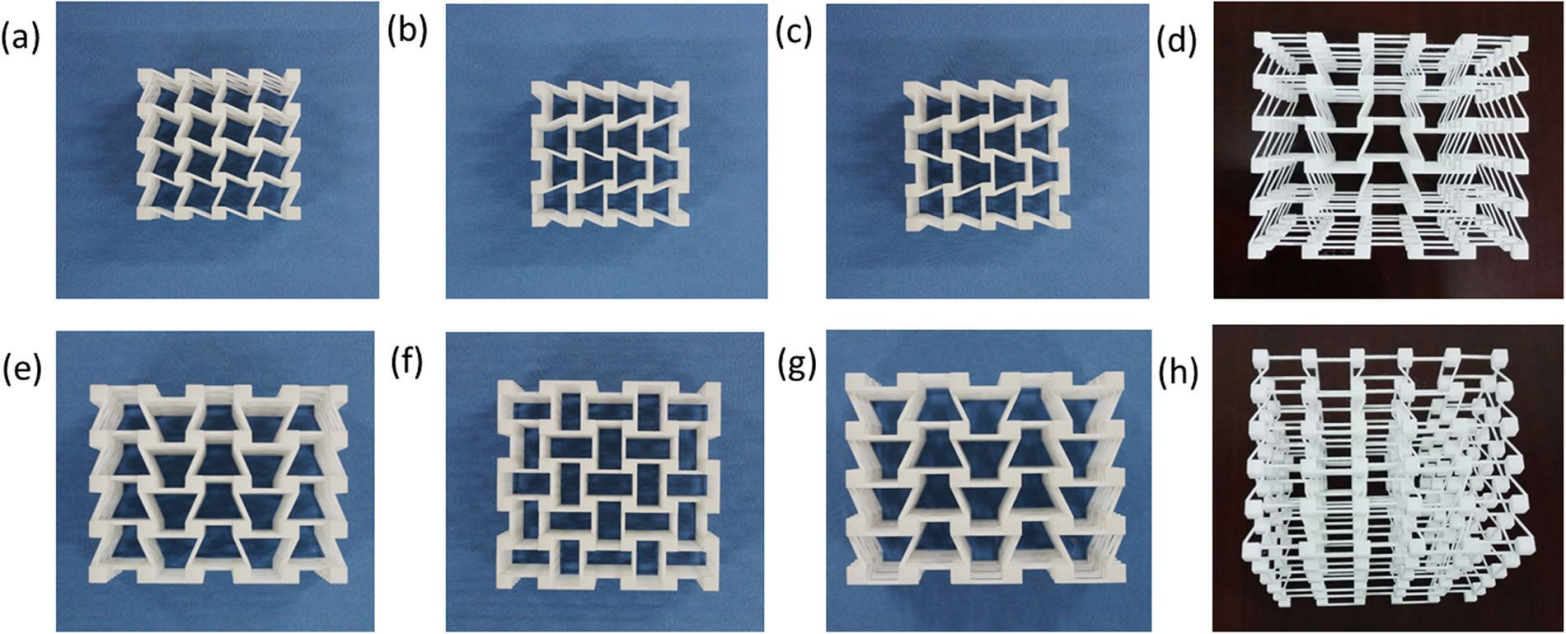
(**a**–**d**) x-y, y-z, z-x and stereo views of as-fabricated chiral- chiral- antichiral metamaterials; (**e**–**h**) x-y, y-z, z-x and perspective view of as-fabricated chiral- antichiral- antichiral metamaterials.

In this subsection, two series of 3D chiral metamaterials with different geometrical parameters are designed, namely Type A and Type B, respectively. The geometrical parameters of these designed two types of 3D chiral metamaterials are shown in Table 1. The two series of chiral metamaterials are fabricated with nylon powder selected laser sintering (SLS) 3D printer @ BMF Material Technology Inc. in Guang Dong Province of China, and the spatial resolution is 8 μm along x, y and z directions. Finally, Type A and Type B samples are fabricated for these two types of 3D chiral metamaterials, and the as-fabricated Type A samples are shown in Fig. 2.

**Table 1:** Geometrical parameters for the two types of 3D chiral metamaterials samples (ligament length L; node width d; ligament thickness t; Number of nodes along x direction N_x_; Number of nodes along y direction N_y_; Number of nodes along z direction N_z_).

3D chiral metamaterials No.		**L**(mm)	**d**(mm)	**t**(mm)	**N**_**x**_	**N**_**y**_	N_z_
Chiral-chiral-antichiral	A	10	5	1	6	6	6
	B	10	6	1.5	5	5	5
Chiral-antichiral-antichiral	A	12	5	1	6	6	6
	B	10	6	1.5	6	6	6

**Figure 3:**
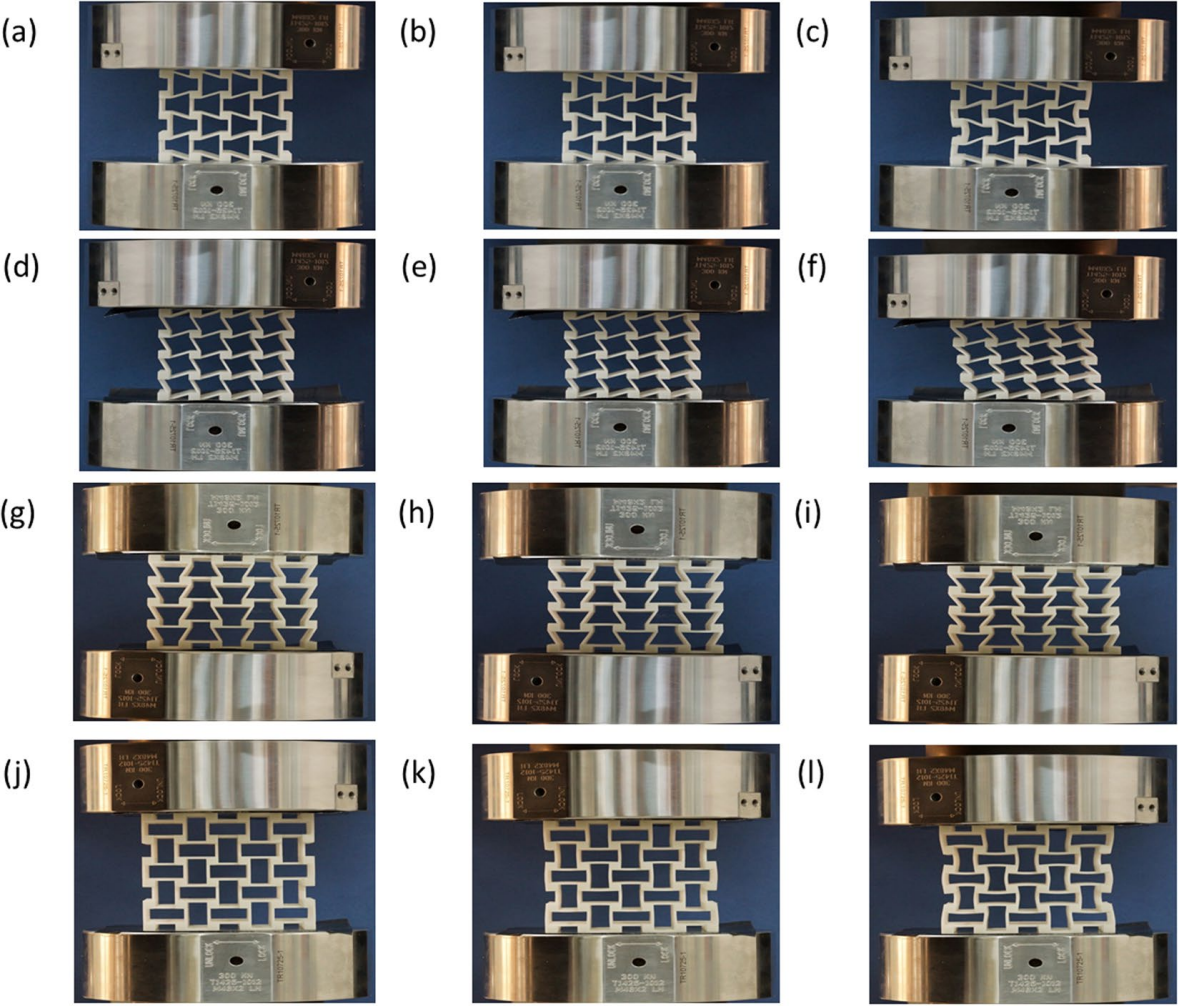
Deformation of 3D chiral- chiral- antichiral metamaterials under uniaxial compression test condition at different compression strain level along z antichiral direction (**a**) ε_z_ = 2%, (b) ε_z_ = 5%, (**c**) ε_z_ = 10%; Deformation of 3D chiral- chiral- antichiral metamaterials under uniaxial compression test condition at different compression strain level along y chiral direction (**d**) ε_y_ = 2%, (**e**) ε_y_ = 5%, (f) ε_y_ = 10%; Deformation of 3D chiral- antichiral- antichiral metamaterials under uniaxial compression test condition at different compression strain level along z chiral direction (**g**) ε_z_ = 2%, (**h**) ε_z_ = 5%, (**i**) ε_z_ = 10%; Deformation of 3D chiral- antichiral- antichiral metamaterials under uniaxial compression test condition at different compression strain level along y antichiral direction (**j**) ε_y_ = 2%, (**k**) ε_y_ = 5%, (**l**) ε_y_ = 10%.

**Figure 4:**
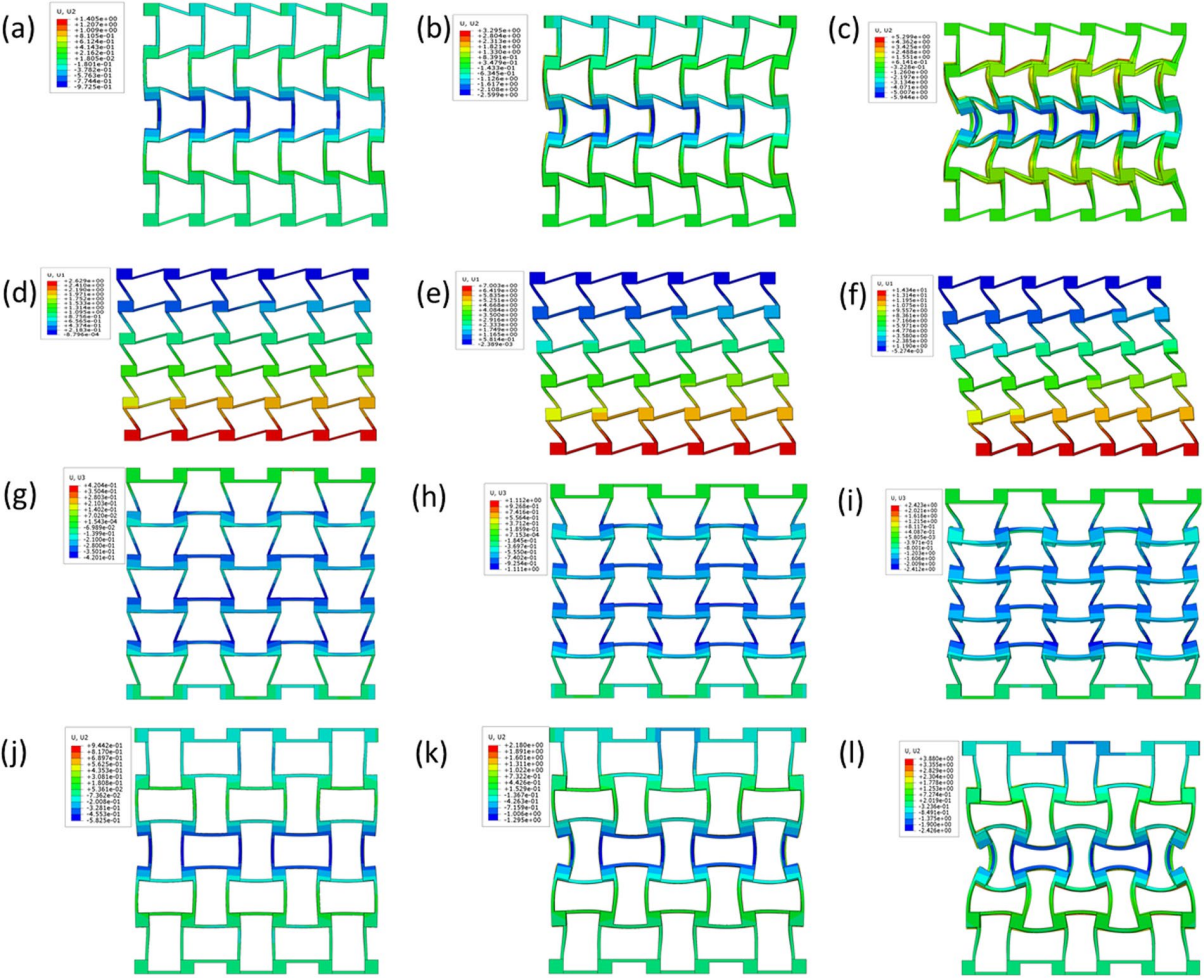
FEA simulated deformation of 3D chiral- chiral- antichiral metamaterials under uniaxial compression test condition at different compression strain level along z antichiral direction (**a**) ε_z_ = 2%, (**b**) ε_z_ = 5%, (**c**) ε_z_ = 10%; FEA simulated deformation of 3D chiral- chiral- antichiral metamaterials under uniaxial compression test condition at different compression strain level along y chiral direction (**d**) ε_y_ = 2%, (e) ε_y_ = 5%, (**f**) ε_y_ = 10%; FEA simulated deformation of 3D chiral- antichiral- antichiral metamaterials under uniaxial compression test condition at different compression strain level along z chiral direction (**g**) ε_z_ = 2%, (**h**) ε_z_ = 5%, (**i**) ε_z_ = 10%; FEA simulated deformation of 3D chiral- antichiral- antichiral metamaterials under uniaxial compression test condition at different compression strain level along y antichiral direction (**j**) ε_y_ = 2%, (**k**) ε_y_ = 5%, (**l**) ε_y_ = 10%.

**Figure 5:**
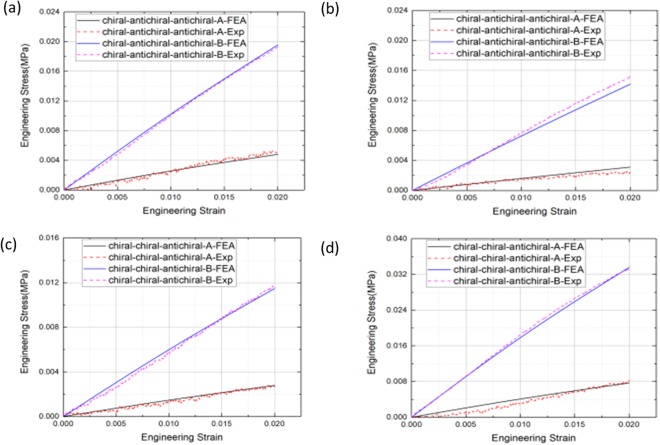
Experimental and FEA simulated strain-stress curves of (**a**) FEA simulated deformation of chiral- chiral- antichiral metamaterials under uniaxial compression test condition until compression strain level ε_z_ = 2% along z antichiral direction; (**b**) FEA simulated deformation of chiral- chiral- antichiral metamaterials under uniaxial compression test condition until compression strain level ε_y_ = 2% along y chiral direction; (**c**) FEA simulated deformation of chiral- antichiral- antichiral metamaterials under uniaxial compression test condition until compression strain level ε_z_ = 2% along z chiral direction; (**d**) FEA simulated deformation of chiral- antichiral- antichiral metamaterials under uniaxial compression test condition until compression strain level ε_y_ = 2% along y antichiral direction.

Before performing compression tests of these two types of 3D chiral metamaterials, the mechanical properties of the SLS nylon material are tested. Totally, 5 uniaxial tensile samples are fabricated, and uniaxial tensile experiments are performed on an Instron®5985 machine at a displacement rate of 1 mm/min. Finally, the average elastic modulus of the 5 as-fabricated tensile samples is:E_s_ = 1021.00 MPa, where the deviation of modulus is: ±0.75 MPa, and the average ultimate strain of the material is ε_max_ = 0.16. After finishing the material properties tests, compression tests of two series of 3D chiral metamaterials are performed, where the loading force, displacement and deformation images during the loading process are recorded, the samples are loaded until compression strain level 10%, and deformation process of these two types of 3D chiral metastructures are shown in Fig. 3. During compression experiments, graphite sheets with quite low friction coefficients (<=0.005) are attached onto the compression contacting surfaces for minimizing the friction force. Finally, relations between axial compression strain and compression stress are generated. Meanwhile, finite element analysis (FEA) simulations of the deformation process of these two series of 3D chiral metamaterials are performed, and comparisons with experimental results are carried out for verification. In all the FEA simulation cases, uniaxial compression displacement loadings are applied on one end of the samples, while the other end of the sample is fixed, where the compression force and axial displacement during the simulation process are recorded until 10% compression strain level, the FEA simulated deformation process of these two types of chiral metamaterials are shown in Fig. 4. The experimental and FEA simulated modulus comparisons for Type A and Type B samples are calculated until 2% compression strain, and the results are shown in Table 2 and Fig. 5.

**Table 2:** Comparison between experimental and FEA simulated modulus (Unit: MPa).

Matematerials type		Chiral-chiral-antichiral		Chiral-antichiral-antichiral	
Loading axis		chiral	antichiral	chiral	antichiral
Type A	Exp	0.105	0.274	0.108	0.196
	FEA	0.139	0.384	0.155	0.240
Type B	Exp	0.559	1.526	0.823	0.981
	FEA	0.574	1.679	0.708	0.977

During the linearly compression process, elastic bending deformation of the ligaments and rotation of circular nodes are dominant, the chiral ligaments form full-wave deformation mode, while the antichiral ligaments form half-wave deformation mode. As shown in Figs 3(a–c) and 4(a–c), the 3D chiral- chiral- antichiral metamaterials was compressed along the antichiral direction, the chiral ligaments form full-wave deformation mode, and the anti-tetrachiral ligaments form half-wave deformation mode, demonstrating auxetic deformation behaviors. As shown in Figs 3(d–f) and 4(d–f), the 3D chiral- chiral- antichiral metamaterials was compressed along the chiral direction, the chiral ligaments form full-wave deformation mode. As shown in Figs 3 (g–i) and 4 (g–i), the 3D chiral- antichiral- antichiral metamaterials was compressed along the chiral direction, the chiral ligaments form full-wave deformation mode, and the anti-tetrachiral ligaments form half-wave deformation mode, demonstrating auxetic deformation behaviors. As shown in Figs 3 (j–l) and 4 (j–l), the 3D chiral- antichiral- antichiral metamaterials was compressed along the antichiral direction, and the anti-tetrachiral ligaments form half-wave deformation mode, demonstrating auxetic deformation behaviors. It can be seen from Figs 3 and 4 that: when these two types of chiral metamaterials are compressed along different loading directions, novel unique deformation mechanisms such as: compression-shearing deformation mechanism and auxetic deformation mechanism can be generated.

### Uniaxial tensile tests of 3D chiral metamaterials

**Figure 6:**
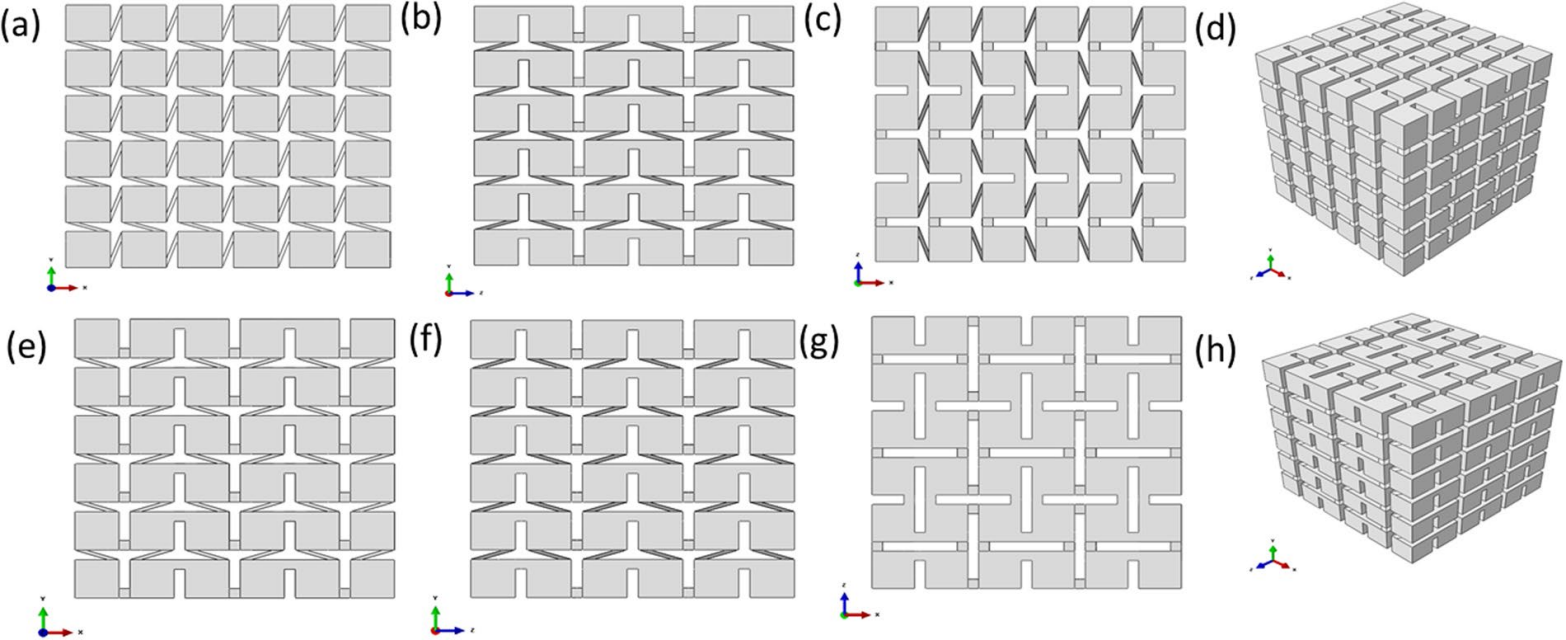
The x-y, y-z, z-x and stereo views of the architected 3D chiral matamaterials (**a**), (**b**), (**c**) and (**d**) chiral- chiral- antichiral metamaterials; (**e**), (**f**), (**g**) and (**h**) chiral- antichiral- antichiral metamaterials.

**Figure 7:**
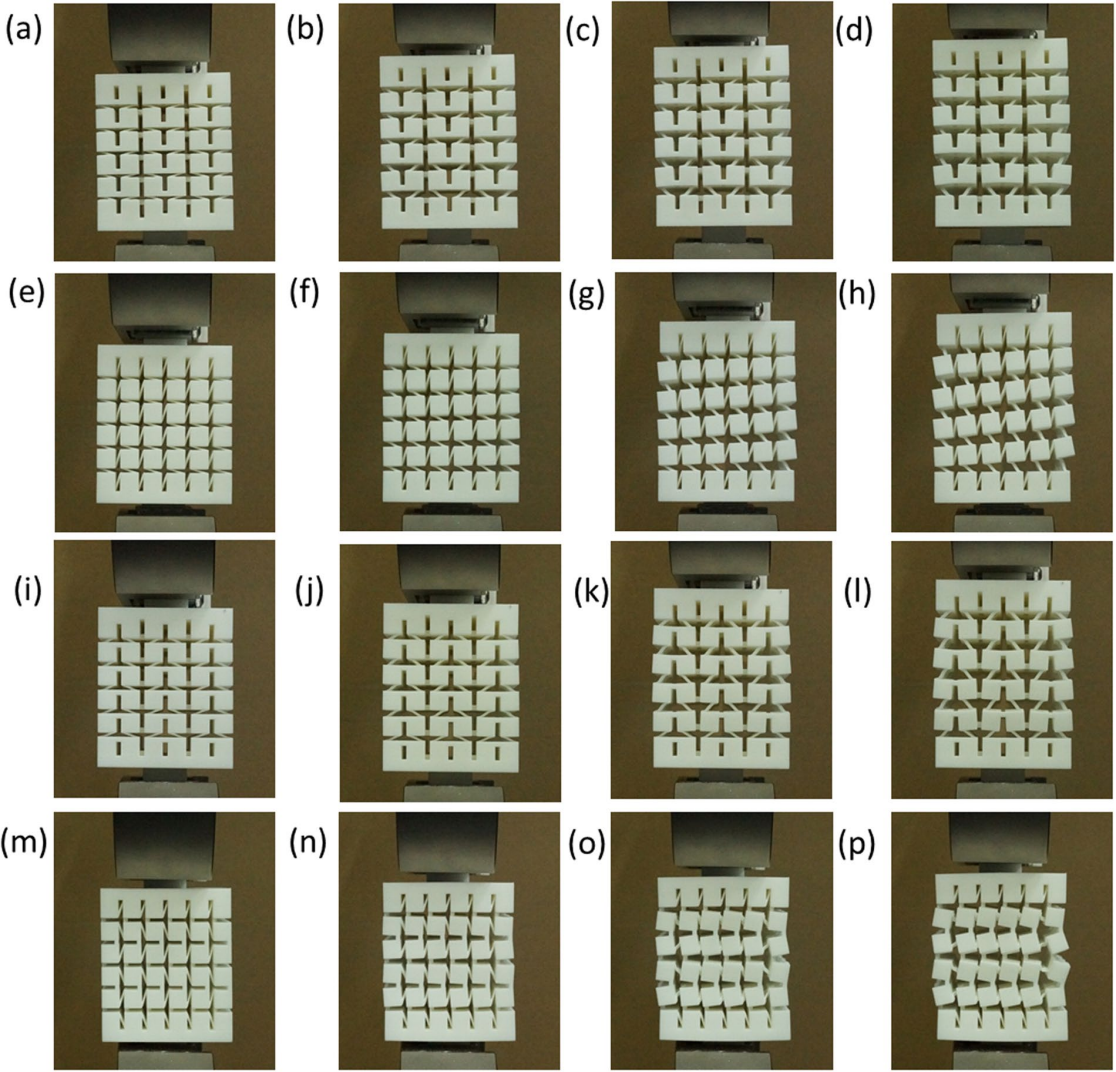
Deformation of chiral- chiral- antichiral metamaterials under uniaxial tensile test condition at different tensile strain level along z antichiral direction (**a**) ε_z_ = 0.0%, (**b**) ε_z_ = 10%, (**c**) ε_z_ = 14%, (**d**) ε_z_ = 18.9%; Deformation of chiral- chiral- antichiral metamaterials under uniaxial tensile test condition at different tensile strain level along y chiral direction (**e**) ε_y_ = 0.0%, (**f**) ε_y_ = 6.7%, (**g**) ε_y_ = 14.6%, (**h**) ε_y_ = 20.5%; Deformation of chiral- antichiral- antichiral metamaterials under uniaxial tensile test condition at different tensile strain level along z chiral direction (**i**) ε_z_ = 4.8%, (**j**) ε_z_ = 4.6%, (**k**) ε_z_ = 11.5%, (**l**) ε_z_ = 17.2%; Deformation of chiral- antichiral- antichiral metamaterials under uniaxial tensile test condition at different tensile strain level along y antichiral direction (**m**) ε_y_ = 0.0%, (**n**) ε_y_ = 3.3%, (**o**) ε_y_ = 7.2%, (**p**) ε_y_ = 9.1%.

In this subsection, uniaxial tensile tests are performed for exploring the deformation mechanisms of these two types of 3D chiral metamaterials. The geometrical parameters of designed two types of 3D chiral metamaterials are shown in Table 3. As shown in Fig. 6, these two types of chiral metamaterials are fabricated with nylon powder selected laser sintering (SLS) 3D printer @ BMF Material Technology Inc. in GuangDong Province of China. Afterwards, *in-situ* tensile tests of 3D chiral metamaterials are performed, where the loading force, displacement and deformation images during the loading process are recorded, and deformation process of these two types of 3D chiral metastructures are shown in Fig. 7. During the linearly straining process, elastic bending deformation of the ligaments and rotation of circular nodes are dominant, the chiral ligaments form full-wave deformation mode, while the antichiral ligaments form half-wave deformation mode. Figure 7 (a–d) show the y-z view of the 3D chiral- chiral- antichiral metamaterials strained along the chiral direction at 0.0%, 10.0%, 14.0% and 18.9% level, and Fig. 7 (e–h) show the x-z view of the 3D chiral- chiral- antichiral metamaterials strained along the chiral direction at 0.0%, 6.7%, 14.6% and 20.5% level. Figure 7 (i–l) show the y-z view of the 3D chiral- chiral- antichiral metamaterials strained along the chiral direction at 0.0%, 3.3%, 7.2% and 9.1% level, and Fig. 7 (m–p) show the z-x view of the 3D chiral- chiral- antichiral metamaterials strained along the antichiral direction at 0.0%, 4.6%, 11.5% and 17.2% level.

**Table 3:** Geometrical parameters for the two types of 3D chiral metamaterials samples (ligament length L; node width d; ligament thickness t; Number of nodes along x direction N_x_; Number of nodes along y direction N_y_; Number of nodes along z direction N_z_).

3D chiral metamaterials No.	**L**(mm)	**d**(mm)	**t**(mm)	**N**_**x**_	**N**_**y**_	**N**_**z**_
Chiral-chiral-antichiral	2	8	2	6	6	6
Chiral-antichiral-antichiral	2	8	2	6	6	6

**Figure 8:**
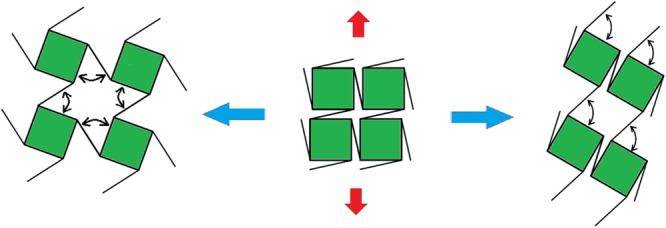
The competition deformation mechanisms: auxetic rotating mechanism without side deformation restriction, and shear deformation due to side deformation restriction.

As to the deformation mechanisms of these two types of 3D chiral metamaterials, rotation of the cubic chiral nodes and bending of ligaments are predominant. (1) As to the 3D chiral- antichiral- antichiral metamaterials, compression-shrinkage and tensile-expanding deformation mechanisms are dominant, where the rotation and bending of chiral-chiral and chiral-antichiral ligament-node pairs will results in auxetic deformation behaviors; (2) As to 3D chiral-chiral-antichiral metamaterials, which can be viewed as cubic solid medium cut out with four-pointed planar star pore systems from two perpendicular directions, where the horizontally inclined ligaments undergo a higher degree of flexural deformation than their vertically inclined counterparts, meaning the rotational symmetry of order four is not being preserved. As shown in Fig. 8, two main competing simplified deformation mechanisms are identified for the chiral-chiral structures of chiral-chiral-antichiral metamaterials, (a) the auxetic rotation deformation mechanism, where both ligaments and nodes rotate by the same amount, producing a negative Poisson ratio −1; (b) the shear-directed deformation mechanism results in a non-zero shear coefficient and a Poisson’s ratio of zero^51,52^. In the compression-shearing experiments shown in Fig. 3 (d–f), graphite sheets with quite low friction coefficients (<=0.005) are attached onto the compression contacting surfaces for minimizing the friction force, and side shearing deformation mechanism is predominant resulting in zero Poisson’s ratio. In the *in-situ* uniaxial tensile experimental tests shown in Fig. 7 (e–h), the auxetic rotating mechanism becomes more active due to the restriction of shear deformation imposed by the uniaxial loading boundary conditions, and thus the system has an overall negative Poisson’s ratio. As tensile strain increases, the Poisson’s ratio gradually becomes even more negative as the rotation of nodes becomes more predominant, resulting in deformation mechanism from shearing deformation dominant towards auxetic rotating deformation dominant.

With the progress of micro- and nano- manufacturing techniques, the proposed 3D chiral metamaterials show promising performances for future industrial applications, such as: nano chiral metatllic glass with extensive hardening and large ductility, sound absorption and vibration attenuation metamaterials, morphing structures, optical chiral metamaterials, shape memory actuators and biomechanical devices.

## Conclusions

In this paper, two types of innovative 3D chiral metamaterials are proposed, namely chiral- chiral- antichiral and chiral- antichiral- antichiral metamaterials. Firstly, two series of 3D chiral metamaterials with different geometrical parameters are designed, and the 3D chiral metamaterials samples are fabricated with Selective Laser Sintering (SLS) nylon sintering techniques, and *in-situ* compression test are performed for studying the mechanical properties and deformation mechanisms of 3D chiral metamaterials. Secondly, *in-situ* uniaxial tensile tests are performed for exploring the competitions between two main types of deformation mechanisms: shearing deformation and auxetic rotating deformation of chiral structures.

The proposed 3D chiral metamaterials represents a series of metamaterials with robust microstructures design feasibilities. With the progress of micro- and nano- manufacturing techniques, the proposed 3D chiral metamaterials show promising performances for future industrial applications, such as: nano chiral metatllic glass with extensive hardening and large ductility, sound absorption and vibration attenuation metamaterials, morphing structures, optical chiral metamaterials, shape memory actuators and biomechanical devices.
